# The Role of Morphological Structure in Determining the Optimal Viewing Position During Visual Word Recognition in Beginning Readers

**DOI:** 10.3390/children11121465

**Published:** 2024-11-29

**Authors:** Stéphanie Ducrot, Séverine Casalis

**Affiliations:** 1 Aix-Marseille Université, CNRS, LPL, UMR 7309, 13100 Aix-en-Provence, France; 2 Univ. Lille, CNRS, UMR 9193-SCALab-Sciences Cognitives et Sciences Affectives, F-59000 Lille, France; severine.casalis@univ-lille.fr

**Keywords:** reading, children, viewing position, length, morphological structure

## Abstract

Background/Objectives: The present study examines the role of morphemic units in the initial word recognition stage among beginning readers. We assess whether and to what extent sublexical units, such as morphemes, are used in processing French words and how their use varies with reading proficiency. Methods: Two experiments were conducted to investigate the perceptual and morphological effects on the recognition of words presented in central vision, using a variable-viewing-position technique. To explore changes during elementary school years, we tested children from the second and fourth grades, as well as adult readers. Results: The percentage of correct word identification was highest near the center of the word, indicating an optimal viewing position for all three participant groups. Viewing position effects were modulated by age and the properties of the stimuli (length and morphological structure). Experiment 1 demonstrated that lexical decisions are influenced by morphological structure to a decreasing extent as reading skill develops. Experiment 2 revealed that morphological processing in children primarily relies on the orthographic information provided by morphemes (surface morphology), whereas proficient readers process morphological information at a more abstract level, exhibiting a genuine morphological-facilitation effect. Conclusions: Overall, our study strongly indicates that morphemic units play a crucial role in the initial stage of word identification in early reading development. This conclusion aligns with the “word and affix” model, which posits that morphological representations become increasingly independent of orthography as reading ability and word exposure improve.

## 1. Introduction

Although reading is rapid and automatic in skilled readers, this ability only emerges in beginning readers through the complex and demanding interplay between perceptual and linguistic processes. The limitations of the visual system significantly constrain how accurately and quickly words can be recognized. Research has shown that perceptual processes may interact with reading efficiency, as fixating slightly to the left of a word’s center can enhance word recognition (see [1,2]), and this optimal fixation position may vary according to the lexical structure of the word. There is an ongoing debate regarding the functional units involved in visual word recognition, especially for beginning readers. The study presented here focuses on the processes that underlie the recognition of visually presented isolated words. Specifically, our experimental work examines how perceptual and lexical factors influence word recognition throughout reading development. We also examine whether sublexical units, such as morphemes, can be encoded as perceptual processing units for beginning readers, thus mediating lexical access and enhancing word recognition.

### 1.1. Learning to Read and Morphological Information

Various factors contribute to the speed of word processing. Research indicates that children generally read short words more quickly than long ones (e.g., [3]), and they tend to recognize frequent words faster than infrequent ones (e.g., [3,4]). However, the impact of word length on reading speed diminishes with age (e.g., [5]). This finding suggests that developing readers shift towards using larger processing units, such as syllables and morphemes, when transitioning from letter-by-letter decoding to proficient and fluent word recognition [6]. In proficient readers, morphemes—defined as the smallest units of meaning, including both roots and affixes—function as critical processing units during the word recognition process. It is widely acknowledged that skilled readers rapidly extract morphological information from written text (see [7,8] for reviews). In children, several studies have shown the importance of morphemic units in reading acquisition (e.g., [4,9,10,11,12,13,14,15,16]; for a review, see [6,17]). Ref. [18] evidenced that children read suffixed words (e.g., lucky) more accurately than control words ending with a suffix-like ending (e.g., pretty). Interestingly, similar effects have been found in Italian, a transparent orthography language. Ref. [19] found that Italian children were faster and more accurate in reading suffixed words (as compared to matched simple control words). Effects of base frequency have also been reported in both languages. Ref. [20] found that children from grades 4 to 6 read low-frequency complex words faster and more accurately when the bases were of high rather than low frequency (see [13] for comparable results in Italian). In visual word recognition, several studies have demonstrated sensitivity to morphological structure across languages in developing readers as young as 7 years old [4,11,15,21,22]. Furthermore, studies that employed the masked priming paradigm to investigate the development of morphological processing in children have produced more variable results. The authors of [14] reported equal priming effects across truly morphological and pseudomorphological conditions in French-speaking 3rd, 5th, and 7th graders, providing evidence for form-based morphological processing that may be operational even in the youngest readers. However, several studies involving English-, German-, or Spanish-speaking children have failed to find evidence for morpho-orthographic priming in primary school children [22,23,24,25], indicating cross-linguistic differences in the efficiency of morphological processing (see [26] for converging evidence that French-speaking children acquire morpho-orthographic decomposition mechanisms sooner than German children). The influence of morphological effects is not confined to priming studies and lexical decision tasks; it also plays a significant role in natural reading contexts. Numerous eye-tracking studies have demonstrated that the way we read sentences is influenced by the syllabic (e.g., [24,25,26,27,28,29]) and morphemic structure of words (e.g., [30,31,32,33,34]).

While substantial evidence suggests that morphemes function as processing units during multimorphemic word processing (e.g., [35,36]), some findings suggest that morphemic units may be activated post-lexically, only subsequent to the activation of lexical representations (e.g., [37]). However, as suggested by [38], morphological effects are more pervasive for children who are not at the endpoint of reading development. Most of the above-mentioned studies used naming and lexical decision tasks. The precise contribution of morphology is not yet completely clear. Because naming relies on phonological aspects and because post-lexical aspects contribute to lexical decisions, additional information is needed to examine whether morphological information contributes to the early stages of word recognition. For this purpose, we will focus on several perceptual constraints in visual word recognition.

### 1.2. The OVP Effect

One of the main results obtained when an experimental manipulation forces participants to fixate a stimulus word at a specific position—which is the focus of interest of the present study—is the optimal viewing position (OVP) effect. The OVP effect indicates “how the initial horizontal placement of the eyes in a word constrains its identification and the subsequent eye movement pattern” (for comprehensive reviews, see [39,40]). This effect refers to the well-known finding that the ease with which printed words are recognized depends on the location where the eyes first fixate (e.g., [1,2,41]). Studies indicate that recognition performance peaks when fixation occurs slightly to the left of the center of a word (e.g., [1,2,42]). The defining characteristics of the OVP effect include not only enhanced performance when fixating on the center of a word compared to its edges but also a notable left–right asymmetry. The resulting function tends to be a J-shape, centered left of the midpoint and asymmetrically skewed in favor of fixations directed towards the start of the word. The effects of fixating outside the OVP have been widely studied (for a review, see [40]). A consistent finding, known as the refixation OVP effect, shows that the frequency of refixing a word—making an additional fixation after the initial one—is lowest when the initial fixation is located at the center of the word (e.g., [43]). The refixation OVP effect has also been observed in continuous reading [41,42]. As reported by [44], this pattern holds true even beyond individual words’ boundaries when presented in parafoveal vision. Typically, the peak or trough of the OVP curves tends to be slightly shifted to the left of the center of words, regardless of their length. However, it should be noted that the asymmetry of these curves tends to increase with longer words, resulting in gradually steeper slopes for the right compared to the left half of the curves. The OVP typically emerges early in the reading acquisition process, usually becoming apparent by the end of the first year of reading instruction [45,46].

Several hypotheses have been proposed to explain this OVP effect. It is generally agreed that the major driving factor is the decrease in visual acuity as a function of distance from fixation, with letters viewed centrally benefiting from a higher resolution than those further from fixation [47]. This constraint predicts that words are recognized most efficiently when fixated at their very center, as fixations at this location maximize the number of letters that project to high-acuity retinal regions. The typical leftward asymmetry of the OVP function can be attributed to different and complementary factors. The first factor is the functional asymmetry related to language processing lateralization, which is typically dominant in the left hemisphere for most readers (e.g., [48,49,50]). Ref. [50] demonstrates that readers who are left-hemisphere-dominant exhibit faster word recognition when fixating more toward the left side of a word compared to their right-hemisphere-dominant peers. Additionally, the visibility of the component letters in a word, along with the informational content they convey, may further contribute to the observed asymmetry in the OVP [51,52]. Moreover, reading habits and perceptual learning can also influence the shape and asymmetry of the OVP curve [39,48]. According to the perceptual learning perspective, optimal word recognition occurs when eye fixation is at the location where the eyes naturally prefer to land, typically between the beginning and the middle of the word [53,54]. Adults who read from left to right demonstrate enhanced word identification abilities within the central or right-to-central part of their visual field as this aligns with their adaptive reading patterns developed during the learning process ([55]; but for a different point of view, see [46,56]).

Another factor to consider is that linguistic information specific to word structure might play a significant role in initial fixation location effects. It has been suggested that the first half of a word often contains more unique linguistic cues that help distinguish one word from similar ones, making the beginning of the word a stronger lexical constraint and, thus, a better source for word recognition (e.g., in French, [1], and, in English, [57]). Ref. [51]’s findings support this idea, showing that the functions of mean ambiguities calculated as a function of fixation position closely mirrored previously documented viewing position (VP) functions. If the center of words is an optimal location for word identification, it is not solely due to the increased number of letters that can be extracted from that position; rather, it is also because the extracted letters are typically associated with a smaller subset of words in the lexicon. Interestingly, ref. [52] showed that a constraint measure derived from the relative positional encoding of letters yields fairly precise predictions for VP functions. Given that in languages like English and French, the initial letter represents the most significant source of information [58,59], it follows that fixations occurring between the onset and the midpoint of a word can maximize the total information extracted from all the letters within that word. Several findings have further supported this lexical hypothesis by showing subtle variations in the location of the optimum of OVP curves with the informativeness of the words’ letters. For instance, the distribution of information within a word modulates its OVP, such that it is typically to the left of the word’s middle for words with rare initial trigrams and moves closer to the middle for words with rare final trigrams [44]. Furthermore, ref. [60] demonstrated that when the initial fixation location was controlled, subsequent refixations were oriented toward letters that provide critical orthographic cues essential for distinguishing a word from its close neighbors. Ref. [60] reported that when the initial fixation location was controlled, refixations were directed towards letters containing critical orthographic information for distinguishing a word from similar ones. Additionally, low-frequency words demonstrate diminished processing efficiency when fixated away from the OVP [2], and the leftward advantage for initial fixation is modulated by the neighborhood characteristics of the target word [61].

Furthermore, the morphological structure of words significantly influences the OVP effect in non-concatenative languages such as Hebrew and Arabic [62,63,64]. In both Arabic and Hebrew, the OVP is typically located at the center of the word [63,65]. A study comparing the OVP in French and Hebrew—written from right to left—found that derivational morphological constraints play a crucial role in determining the optimal viewing location [62]. Derivational morphology is how suffixed/prefixed words can be derived from a base word (e.g., view) through morphological processes such as adding a suffix (er in viewer) and/or a prefix (pre in preview) according to morphological construction rules. When the word’s root is placed at the beginning, there tends to be an asymmetric distribution favoring the right (first) half of the word, reflecting left visual field (LVF) superiority. This LVF advantage diminished and was even reversed when the root letters were centered within the word. This result is consistent with the findings of [63], the authors of which showed that the VP curves in Arabic vary based on morphological structure. Prefixed words showed a leftward advantage (favoring word endings), while suffixed words showed a rightward advantage (favoring word beginnings). Despite these insights, empirical research examining how the internal structure of words might modulate the peak of the VP function, as opposed to more overarching phenomena like reading direction, visual span, or perceptual learning, remains scarce [53,66].

This study investigates the influence of morphemic units on the initial stages of word recognition in beginning readers. We assess whether and to what extent sublexical units, such as morphemes, are used in processing French words and whether the use of these sublexical units changes with reading proficiency. The OVP paradigm provides a valuable framework for examining the perceptual processing of morphemic units, as the OVP may differ as a function of reading efficiency and lexical factors, including word frequency and word structure. We investigated the hypothesis that the morphological structure of word stimuli can influence the shape of the viewing position function. If morphemes are indeed critical units in the early stages of reading acquisition, as suggested by previous studies, we expect that even the earliest processing stages—assessed by a viewing position manipulation—could be affected by the morphological structure of words. This raises the question of whether it is important for the reading system to identify the location of a word’s root, potentially leading to a shift in the VP function peak towards the root’s location. To examine the potential variations in processing related to the reading experience, we conducted experiments with participants at different proficiency levels, including second and fourth graders and adult readers.

The research was conducted per the Declaration of Helsinki [67] and received approval from the Comité de Protection des Personnes ethics committee at Aix-Marseille University (ANR project LECT MORPHO). Before participants could be enrolled in the study, we obtained written informed consent from both the participants and their legal guardians.

## 2. Experiment 1

The first experiment explored how the properties of stimuli, specifically the length and morphological structure, and reading proficiency influence eye movement behavior within words, particularly the OVP effect. To assess the impact of morphological structure, the study compared suffixed words (combinations of a root morpheme and a suffix) with monomorphemic words.

### 2.1. Method

Participants. A total of 40 children—21 s-grade children (11 girls and 10 boys, mean age = 7.7 years, range = 7–8), 19 fourth-grade children (10 girls and 9 boys, mean age = 9.7 years, range = 9–10), and 25 adult participants (18 girls and 7 boys, age range of 18 to 25 years) took part voluntarily in the experiment. The adult participants were all students at Aix-Marseille University (France). The children were recruited from an elementary school in Lille, France. Their parents gave informed consent, and the board of education agreed to the study. All participants had normal or corrected-to-normal vision and spoke French as their native language. To ensure the representativeness of our sample, the reading ability (speed and accuracy combined) of all the children was tested using the standardized French reading test “L’Alouette” (The Alouette test is the most used test in France to evaluate reading efficiency. It consists of sentences that are syntactically correct but semantically impoverished, ensuring only a focused evaluation of decoding skills.) [68,69]. The mean reading age in second grade (M = 8 years 0 months, SD = 8 months) and fourth grade (M = 10 years 1 month, SD = 9 months) did not differ significantly from the corresponding chronological age (ts < 1). The test took place at the end of the academic year. All participants had normal or corrected-to-normal vision and spoke French as their native language. Those who had sensory, neurological, or any other issues typically considered as exclusion criteria for learning disabilities were not included in the study.

Materials, design, and stimuli. A total of 200 words were used. The targets were divided into two subsets: monomorphemic words (e.g., “cerise”/“cherry”) and morphologically complex words (i.e., whose meaning corresponds to the association of the respective root and suffix meanings (e.g., “rasoir”/“razor” = “raser” + “_oir”)). The words were selected from Manulex4 [70]. Half were short, and half were long; 5- to 7-letter words (M = 6) were classified as short, and 9- to 11-letter words (M = 10) as long. The word frequency (mean printed frequency of 20 occurrences per million) and suffix length (3 characters long) were controlled.

**Figure 1:**
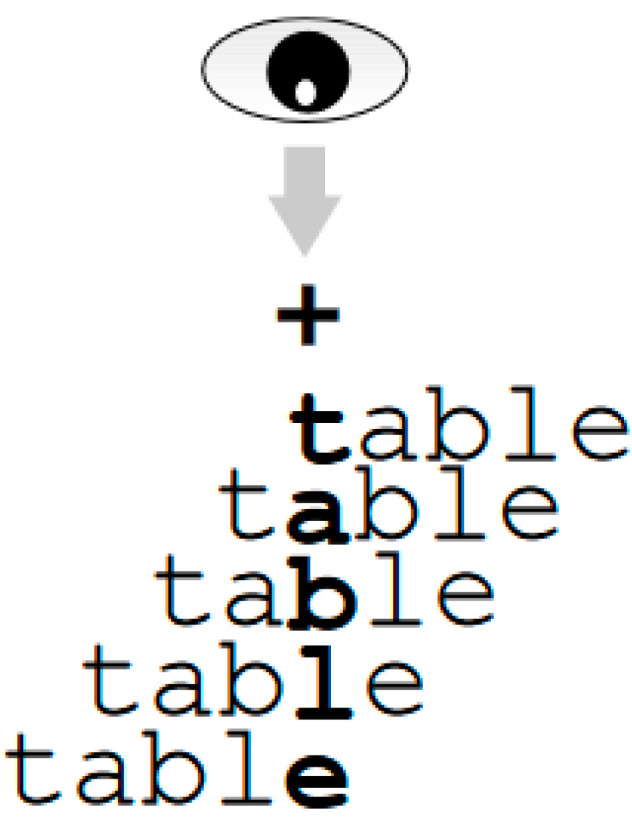
Example of how the initial fixation position was manipulated in the optimal viewing-position paradigm (adapted from [71]).

Apparatus and procedure. The procedure implemented was identical to that described in the study described in [46]. All children were tested individually at school. Words were displayed in white lowercase letters against a black background in 24-point Courier New font, using a 14-inch color monitor at a resolution of 1024 × 768. Participants were seated 60 cm from the screen. At this distance, one letter subtended a visual angle of 0.5°. Each word was divided into five zones of equal width (i.e., 1.2 letters wide for six-letter words and 2 letters wide for ten-letter words). Words were presented so that subjects initially fixated on the center of each zone (hereafter called positions P1, P2, P3, P4, and P5). For example, in the P5 condition, stimuli were presented with their last letters on the central fixation point. Across all participants, each word was seen from all five fixation positions. The target exposure time was determined individually for each participant, depending on his/her correct identification score in a 24-item training session (in which we looked for the presentation duration that produced scores ranging between 50 and 75% correct four-letter word identification, i.e., about 60 ms for adults, and 125 and 200 ms for fourth- and second-grade children (Selecting younger children (e.g., at the end of 1st grade) would not have permitted us to use a display duration of words below the latency of a saccade to ensure that children would have a single fixation on long words), respectively. Each trial consisted of the following sequence of events (see Figure 1). First, participants had to fixate on the cross displayed in the middle of the screen and not move their eyes. The importance of continuing to focus on this point was stressed repeatedly. Then, 500 ms later, the fixation point was replaced by a target word that was displayed on the screen for the duration previously determined for that particular child. The duration used was brief enough to discourage eye movements [40]. The word was displaced laterally with respect to the fixation point according to its position condition. Then, the word was replaced by a backward mask, which consisted of a string of hashes. The task was to identify (name) the target word. If this was not possible, participants were asked to report as many letters as possible in the correct position if this was impossible. The experimenter manually recorded each participant’s response. The mask remained on the screen until the experimenter pressed the space bar to trigger the next trial. A 24-item practice session was held in advance. This was followed by a single experimental block of 200 trials composed of suffixed and control words. All participants were given a break halfway through the experiment, and additional breaks were given whenever required. The experiment lasted approximately half an hour for the second graders and less than 20 min for the adults.

We performed the analysis on response accuracy using generalized linear mixed-effect modeling, with the function glmer of the package lme4 [72]. A likelihood ratio test assessed the significance of individual factors and their interactions. This method helps determine whether the model fit changed significantly when adding a factor or an interaction. The factors of interest included age (2nd grade, 4th grade, adult), type of stimulus (suffixed words and control words), length (short vs. long words), and fixation position (P1, P2, P3, P4, and P5). The results are summarized in Table 1.

### 2.2. Results and Discussion

The random structure included only by-participant random intercepts, given that accuracy scores were calculated on all items. Adding age significantly improved the model, [χ^2^ = 18.43, *p* < 0.001], reflecting the fact that the younger children made more errors (48.7%) than older children and adults (39.3% and 36% for adults and fourth graders, respectively).

**Figure 2:**
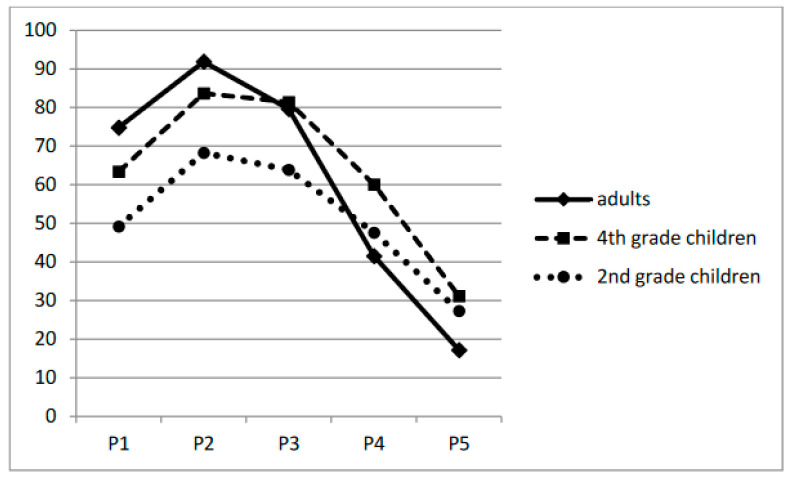
Percentage of correct word identification as a function of the initial fixation position and age in Experiment 1.

The effect of position was also significant: adding position as a fixed effect resulted in a significant improvement in fit [χ^2^ = 1622.20, *p* < 0.001]. As can be seen in Figure 2, the location in the words where the curves increased to their maximum was to the left of the target’s center, thus suggesting an OVP and confirming the relevance of a simplified version of the variable VP technique. There was also a significant fixation position by participant group interaction, χ^2^ = 288.22, *p* < 0.001, with a weaker difference between fixating on the beginning and the end of the word (21.9%) for the second graders compared with the other groups (32.2% and 57.6%, for fourth graders and adults, respectively).

**Figure 3:**
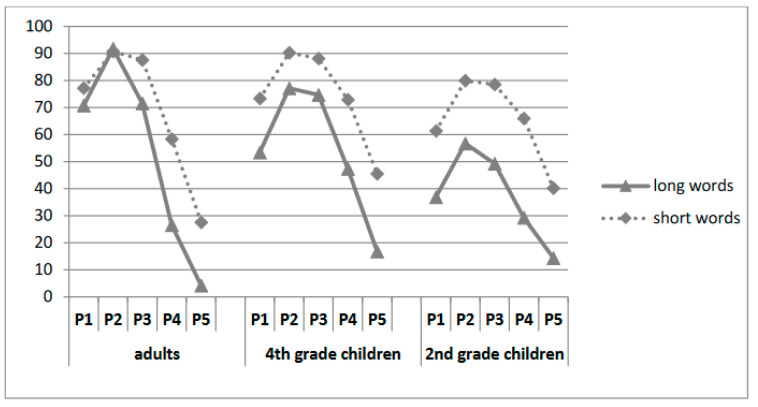
Percentage of correct word identification as a function of the initial fixation position and length for the 3 groups in Experiment 1.

There was also a significant main effect of length: adding length as a fixed effect improved the model fit (χ^2^ = 91.545, *p* < 0.001) with better performance for short (69%) than for long words (48.2%). Moreover, adding the interaction between grade and length significantly improved the fit [χ^2^ = 51.16, *p* < 0.001]: the word length effect was significantly reduced from second to fourth grade and from fourth grade to adults. Most importantly, there was a significant length by fixation-position interaction [χ^2^ = 5.29, *p* = 0.02], indicating that the length effect was larger at unfavorable positions. In line with this, there was a significant length x fixation position x age interaction [χ^2^ = 23.25, *p* < 0.001]. As shown in Figure 3, adults and fourth graders showed reduced length effects for fixations on the left half of the word. In contrast, for beginning readers, the size of the length effect was similar for all positions.

There was no main effect of word structure. However, adding the interaction between grade and word structure improved the model fit [χ^2^ = 21.294, *p* < 0.001]: the word structure was significant in younger children (*p* < 0.001) and disappeared in adults. We also found that the word structure effect was stronger for long words [χ^2^ = 64.404, *p* < 0.001], with better performance for suffixed words in the long word condition.

**Figure 4:**
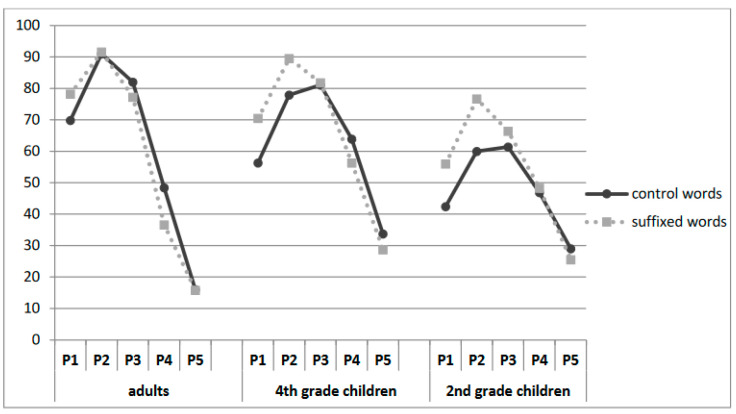
Percentage of correct word identification as a function of the stimulus type and fixation position for the 3 groups in Experiment 1.

Interestingly, there was also a significant word structure by fixation-position interaction [χ^2^ = 69.148, *p* < 0.001], with fixations on the left VPs within the word affecting control words and suffixed words differently. Suffixed words led to higher performances, especially in the initial letter positions (i.e., at critical letter positions corresponding to the root). No difference was observed between control words and suffixed words in P4–P5. As Figure 4 illustrates, the effect was modulated by age [χ^2^ = 6.361, *p* < 0.01], with children exhibiting a greater left-half-VP advantage for the suffixed words in P1-P3 conditions than adult readers.

### 2.3. Discussion

First, an OVP effect was observed, indicating that the likelihood of recognizing a word was greater when the eyes first fixated slightly to the left of the center of the word. This advantage for the left half of the word demonstrates what is known as right visual field (RVF) superiority, a phenomenon that has been observed in languages written from left to right. The OVP effect was influenced by (1) word length: the length effect appeared larger at unfavorable positions; and (2) word structure: morphological information seemed to help more at the beginning of the word, suggesting that OVP effects are partially influenced by morphological processing. These effects were modulated by grade, with a weaker P1/P5 asymmetry for beginning readers (see [46] for a similar result). Our findings for participants with varying reading levels suggest that word identification is influenced by morphological structure to a decreasing extent as reading skills develop. Note that we also observed that the morphological structure effect was stronger for longer words. For all groups, suffixed words were easier to recognize in the case of long words, suggesting that the role of morphology on longer words may be affected, at least in part, by visual constraints of the eye. This pattern of results confirmed that perceptual and lexical factors interact in visual word recognition in children.

## 3. Experiment 2

Experiment 1 demonstrated that beginning readers can directly encode morphemes as perceptual processing units of word recognition. The second experiment aimed to disentangle whether this facilitation was due to the word’s in-depth morphological processing or sensitivity to the morphological surface structure. The morphological effect may reflect a lexical activation of morphologically related words once the base has been identified. Therefore, morphologically complex words are identified more often than control items because the identification of the base automatically activates morphologically complex words that are derived from the base. Alternatively, the facilitation might be limited to a purely formal aspect. If morphemes are perceptual units, then identifying both bases and suffixes might be faster than for control letter groups. In this case, the benefit of morphological words over control words should be observed even without a semantic relationship between the “base”—in other words, the embedded word at the onset of the word—and the whole word. For example, *corn* should facilitate the identification of *corner* (pseudo-suffixed pairs, see [12,16]) as *sing* facilitates the identification of *singer* (suffixed pairs) even though only sing and singer share a morphological link.

### 3.1. Method

Participants. All participants in the study were native French speakers with normal or corrected-to-normal vision and no known reading difficulties. Participation was voluntary, and all participants were unaware of the study’s purpose. The study included 22 adult participants, all Aix-Marseille University students ranging from 19 to 27 years old. Additionally, 49 child participants volunteered from a local school in the Lille area. Among the child participants, 18 were younger children aged 7 to 8 years (comprising 8 girls and 10 boys, with a mean age of 7.8 years and a mean reading age of 8 years and 1 month, SD = 8 months). The remaining 31 were older children aged 9 to 10 years (consisting of 16 girls and 15 boys, with a mean age of 9.1 years and a mean reading age of 9 years and 8 months, SD = 6 months). These children were in the 2nd and 4th grades, respectively. The mean reading age of the children did not differ significantly from the corresponding chronological age (ts < 1). Participants were excluded from the study based on sensory, neurological, or other conditions typically recognized as exclusion criteria for learning disabilities.

Materials. A pool of 135 six-, seven-, and eight-letter words was selected from Manulex [70]. To test for the formal and semantic effects of morphological structure, three categories of items were considered: suffixed words, which were genuine morphologically derived words (e.g., “singer” = “sing” + “er”), pseudo-suffixed words, in which the whole word was not a derived form of the embedded word at the onset (e.g., “corner” = “corn” + “er”), and control words, in which there was no morphological structure at all, embedded root or suffix-like ending. There were 45 items in each category, matched for length and frequency. Words were matched for print frequency (20.44 occurrences per million, ns) but could not be perfectly matched for length (*p* = 0.015) as the morphologically derived words were longer than the pseudo-suffixed and control words (7.27 vs. 7.07 and 6.96 letters, respectively). The print frequency of the bigrams was also controlled.

Apparatus and procedure. Experiment 2 used the same apparatus and procedure as Experiment 1, except for a simplified version of the variable viewing-position technique [73]. As in Experiment 1, each stimulus was divided into five equal-width zones (1.2 letters wide for 6-letter words, 1.4 letters wide for 7-letter words, and 1.6 letters wide for 8-letter words). The stimuli were presented so that participants first fixated on the center of one of the following zones: the leftmost zone, the zone just to the left of the word’s center (referred to as the OVP zone), or the rightmost zone. These fixation positions are designated as P1, P3, and P5, respectively. Each participant viewed every stimulus from all three fixation positions. Participants were allowed short breaks after every 45 trials. A twelve-item training phase was held at the beginning of the session, followed by a single experimental block of 135 trials. As in Experiment 1, the target exposure time was determined individually for each participant, depending on his/her correct identification score in a 24-item training session (i.e., about 60 ms for adults, 150 ms for fourth graders, and 200 ms for second graders). Factors of interest were thus age (adult vs. 4th grade vs. 2nd grade), morphological structure (suffixed vs. pseudo-suffixed vs. control words), and initial fixation position (P1 vs. P3 vs. P5). All factors except age were manipulated within participants. The results are summarized in Table 2.

### 3.2. Results and Discussion

**Figure 5:**
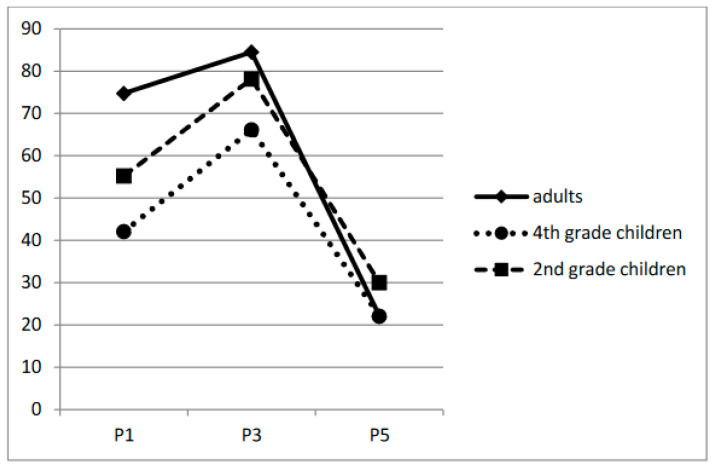
Percentage of correct word identification as a function of the initial fixation position and participant group in Experiment 2.

As for Experiment 1, we performed the analysis on response accuracy using generalized linear mixed-effect modeling, with the function glmer of the package lme4 [72]. The effect of age was significant as adding age significantly improved the model fit [χ^2^ = 24.35, *p* < 0.001], reflecting the fact that the younger children made more errors than the older children and adults (56.54%, 45.5%, and 38.52%, respectively). In addition, there was a main effect of fixation position [χ^2^ = 2387.90, *p* < 0.001] and a significant age by fixation position interaction [χ^2^ = 304.68, *p* < 0.001]. As in Experiment 1, there was a stronger P1/P5 asymmetry for the expert readers (52.6%) compared to the children (25.2% and 20% for grade 4 and grade 2, respectively, Figure 5).

The morphological structure’s main effect was non-significant [χ^2^ = 3.55, ns]. However, it interacted significantly with age [χ^2^ = 53.21, *p* < 0.001]. The effect of morphological structure was significant in both groups of children (*p* < 0.001) but not significant in adult readers. Pairwise comparisons revealed that for children, this effect could be explained by the fact that there was a difference between morphologically complex stimuli (suffixed words and pseudo-suffixed words) and control words (*p* < 0.001) and no difference between suffixed words and pseudo-suffixed words (*p* > 0.05).

Interestingly, the interaction between morphological structure and fixation position improved the model fit [χ^2^ = 51.42, *p* < 0.001], which indicated that, as in Experiment 1, the difference between morphologically complex and control words was more pronounced for the left half of the word (*p* < 0.0001). No difference was observed between suffixed words and pseudo-suffixed words.

#### By-Group Analyses

**Figure 6:**
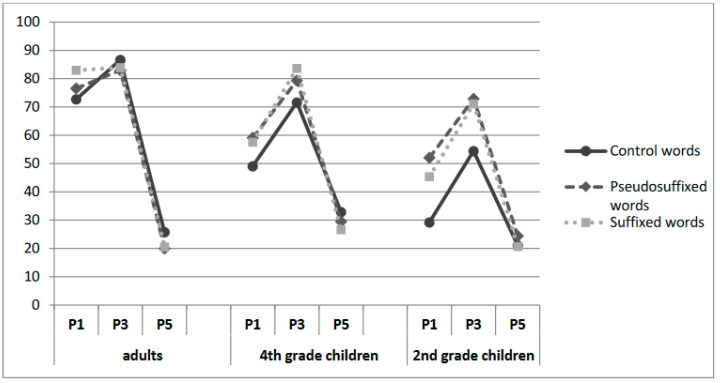
Correct word identification results as a function of the stimulus type and fixation position for the 3 groups (Experiment 2).

Adults. The effect of morphological structure was not significant. Adding position significantly improved the model fit [χ^2^ = 1407, *p* < 0.001], and the interaction between morphological structure and fixation position was significant [χ^2^ = 9.95, *p* = 0.041]. The results are clear-cut, as suggested by Figure 6, with no effect of morphological structure in P3, an advantage of suffixed words in P1 [*p* = 0.0001, with no difference between pseudo-suffixed words and control words], and an advantage of control words in P5 [*p* < 0.01; the difference between suffixed words and pseudo-suffixed words was marginally significant, *p* = 0.082].

Fourth graders. The word structure was not significant. The effect of fixation position significantly improved the model fit [χ^2^ = 890.27, *p* < 0.001], and the interaction between fixation position and morphological structure was significant [χ^2^ = 30.02, *p* < 0.001]. As can be seen in Figure 6, there was a stronger left-half-VP advantage for the suffixed words and pseudo-suffixed words compared to control words. Pairwise comparisons revealed a significant difference between the morphologically complex and control words in P1 (*p* = 0.0002) and P3 (*p* = 0.0002) and no difference between suffixed words and pseudo-suffixed words (*p* > 0.05). However, in P5, the effect was reversed with lower word identification for suffixed words compared to control words (*p* = 0.026). Note that the difference between pseudo-suffixed and suffixed words was non-significant (*p* > 0.05), as was the difference between pseudo-suffixed words and control words.

Second graders. As for older children, there was a significant effect of fixation position [χ^2^ = 453,78, *p* < 0.001] and a significant interaction between the effects of morphological structure and fixation position [χ^2^ = 23.71, *p* < 0.001], with a stronger word-beginning-VP advantage for words with a morphological structure (suffixed words and pseudo-suffixed words) compared to control words. Pairwise comparisons revealed a significant difference between the morphologically complex and control words in P1 (*p* < 0.0001) and in P3 (*p* < 0.0001). However, in P5, no effect of morphological structure was obtained (all *p* > 0.05).

### 3.3. Discussion

The results of Experiment 2 suggest that both VP and morphology influence the OVP in children. This is apparent from the performance observed for each type of word, with suffixed and pseudo-suffixed words significantly helping word recognition. As expected, we found a drop in performance when the fixation point was shifted horizontally from the OVP. Moreover, this decrease seemed to interact with the morphological structure of the word. The word identification score was higher when the initial fixation position corresponded to the root (P1 and P3), without any benefit of an initial fixation position corresponding to the suffix (P5). Since the end of suffixed words and pseudo-suffixed words consisted of relatively uninformative lexical information, the effect was reversed with better performances for control words when older children and adults first fixated on the word’s end, thus suggesting that morphemes are functional and perceptual units of word recognition as soon as grade 4. Considering the lack of differences between suffixed words and pseudo-suffixed words in second and fourth graders, two possibilities must be contemplated: either children are critically sensitive to embedded words, regardless of their morphological properties, or morphological processing in children appears to be based on the orthographic information provided by morphemes (surface morphology). In adults, the results were clear-cut with (1) a specific advantage of suffixed words in P1 and (2) no difference between the three types of words in P3 where VP conditions were optimal. These results thus suggest that expert readers process morphological information at a different level, with a genuine morphological-facilitation effect. They are less sensitive than developing readers to the presence of an “embedded word” and a suffix within a larger word to facilitate its identification.

## 4. General Discussion

The rationale for the current experiments was to investigate the influence of morphemic units in the initial stage of word recognition among beginning readers. We used the OVP paradigm to examine whether morphemes can be directly encoded as perceptual processing units of word recognition by beginning readers. We hypothesized that the peak of the VP function for French words would shift as a function of word properties and participants’ reading proficiency, with the idea that morphemes serve as both functional and perceptual units in the word recognition process. To assess reading skills, we tested second graders and compared their performance with fourth graders and adults.

The results from our two experiments add to the evidence in favor of a functional role for morphology during children’s visual word recognition. We found that as reading skills improved, the influence of morphological structure decreased, suggesting that children are more sensitive to morphological structure than adults. This provides important new evidence from a perceptive identification task, which aligns with a wider array of priming and lexical decision studies (e.g., 14, 18–20, 26). In Experiment 2, we observed no significant difference between suffixed words and pseudo-suffixed words in second and fourth graders. This suggests that the presence of an “embedded word” and a suffix within a longer word helps facilitate word recognition in children. The data also make a significant contribution to the discussion regarding the source of the OVP effect. In both experiments, we found an interaction between VP and morphology. Performance improved when the initial fixation position corresponded to the root of words with a morphological structure (such as suffixed and pseudo-suffixed words) compared to control words. This finding confirms that the lexical properties of words can influence the shape of the VP function. Additionally, our findings support the idea that the asymmetry of the VP function, which peaks to the left of the center, may occur because the beginnings of words generally contain more critical information than their endings [51].

### 4.1. The Role of Lexical Factors in Shaping the OVP Effect

Previous research has suggested that decreased visual acuity with retinal eccentricity contributes to the OVP effects observed in word recognition. Specifically, as the distance of letters from the fixation point increases, visual acuity deteriorates, leading to poor visual information processing [39,41,74,75,76]. When participants were instructed to fixate on either the initial or final letters of words, their ability to recognize those words was impaired due to these visual acuity limitations. Our data corroborate that the probability of successfully identifying the target word decreased when the initial fixation was placed on the beginning or ending of the word rather than to the left of the word center, with a left/right asymmetry, leading to an OVP effect. This left-half advantage indicates RVF superiority, a phenomenon previously documented in languages read from left to right. These results imply that word beginnings may be particularly important in the OVP effect. Earlier research has established that the initial letter of a word conveys substantial information, with knowledge of this first letter proving more beneficial for word identification than that of the final letter [39,57,77,78,79]. Furthermore, we found that these VP effects were modulated by reading grade, revealing a weaker P1/P5 asymmetry among beginning readers (see [46] for similar results).

The OVP curve was influenced by word length, with OVP effects becoming more pronounced for longer words. This occurs because initially fixating on a non-optimal position within a long word significantly hampers word processing when several letters are outside the fovea, as demonstrated by [80]. The stronger OVP effect observed for long words compared to short ones supports the visual acuity account, which posits that these effects underscore the limitations inherent to foveal vision (but see [39,81] for an alternative account, the interhemispheric transfer time account, which also predicts a bigger left-right asymmetry for long words than for short words).

A key finding of our study was that similar to non-concatenative languages like Hebrew and Arabic [62,63,64], the OVP curve was influenced by word structure. Our results indicate that morphological information is particularly beneficial at the beginning of the word, suggesting that OVP effects are, in part, driven by morphological processing, especially regarding the position of the root (see [43,76,80,82] for contrasting evidence regarding the impact of lexical factors on the OVP effect). OVP effects were more pronounced for morphologically complex words than control words, with a stronger left-half-VP advantage for the derived words (suffixed words in Experiment 1 and suffixed and pseudo-suffixed words in Experiment 2). These effects varied based on grade level. It can be argued that the impact of word structure, along with its interactions with other variables, increases under conditions of poorer presentation quality, stimulus attributes, or participants’ reading abilities (for analogous findings in adults, refer to [83]). The final observation in the present study was that the morphological structure effect was stronger for long words. For all groups, suffixed words were easier to recognize in the case of long words, indicating that visual constraints may adjust the role of morphology in the processing of derived words. Word length appears to be a significant modifying factor not solely for derived terms but also for compounds. The authors of [84] demonstrated in continuous reading that the first constituent of a long compound benefits from what they termed “a visual acuity advantage” over the second constituent and the whole word. In contrast, all sublexical and lexical information can be extracted simultaneously with short compounds. This pattern of results confirmed that perceptual and lexical factors interact during visual word recognition in children.

The OVP may thus rely on fine-tuning between word lexical properties, perceptual/attentional factors, and reading experience.

### 4.2. The Role of Morphological Information in Children

This study’s primary question investigated whether and to what extent sublexical units, such as morphemes, are involved in the processing of French words and how the use of these units varies with reading proficiency. Our findings suggest that the morphological structure of words significantly influences the initial stages of word identification, corroborating similar results observed in Arabic [85]. Previous studies have demonstrated that children exhibit sensitivity to the morphological structures of words during the reading process. These studies generally used naming and lexical decision tasks. The results indicated that accessing a base morpheme can facilitate reading morphologically complex words. The contribution of the base to reading a complex word in child reading was also suggested by the base frequency effect found in the study of [20]. All these studies compared morphological words to non-morphological words. Note that non-morphological words sometimes include embedded words at the root place (such as in spinach) of a suffix-like ending (such as in pretty). In [14], the presence of a base and suffix was considered in a systematic way, with a combination of the presence and absence of both embedded words and suffixes. It was found that the presence of a base and/or suffix facilitated word recognition. The authors concluded that “both bases and suffixes have acquired a specific status in the word recognition system, and their presence offers young readers a reliable clue in lexical decisions”.

The results of the present study add new evidence for the contribution of morphemes in developing readers. Two critical aspects were manipulated here. First, word identification was examined through a perceptual identification task rather than a lexical decision task, thus limiting post-lexical or decision effects. Using the OVP task enabled us to locate the morphological effect more precisely in terms of perceptual factors. Second, and importantly, we considered two types of morphological words: suffixed and pseudo-suffixed. While suffixed words refer to a genuine morphological analysis (the category that was considered in the aforementioned studies), the pseudo-suffixed words consist of morpheme-like units, including both bases (roots) and suffixes; however, the pseudo-suffixed form is not derived from the base/root. Thus, *corner* is not derived from *corn*, but its structure includes surface morphological aspects just like in *singer*/*sing*. This key condition enabled us to disentangle the issue of which aspects of morphological structure are relevant in these first stages of word processing. Given the absence of differences between suffixed and pseudo-suffixed conditions, our results may indicate that the locus of the present morphological effect is not semantic—or due to genuine morphological aspects—but reflects a pure surface structure effect. Pseudo-suffixed words, like suffixed words, include both base/root and suffix and it is this effect of morpheme-like units included within a large word that is relevant in the morphological effect. Base/root words appear as “embedded words”, which are salient from a perceptual point of view. Therefore, the processing of letters included in the “embedded word” is sped up compared to a similar string of letters that does not consist of a word. This interpretation is consistent with the fact that morphological effects are stronger in the initial positions (P1–P2) compared to the final positions (P4–P5). At initial positions, participants can choose the base word and benefit from facilitation due to the lexical status of the initial letter string. Alternatively, in the final position, the suffix, i.e., the unit that might be salient in the morphological conditions, is not informative given the number of words ending with a suffix (see [86]). Note that we cannot exclude the possibility that children are not effectively activating morphological units but instead are critically sensitive to embedded words, independent of morphology (see [87]). This account speaks against a true morphological analysis at these ages and suggests that morphological effects in this task may be more epiphenomenal. Participants can activate the embedded word and then, through some inference process, determine a possible ending without needing to have true morphological units.

It is noteworthy that the benefit of morphological information depends on the age group: the younger the participants, the stronger the morphological effect. Again, this result is consistent with our interpretation that the facilitation effect is located in the faster identification of the first letters when they consist of an “embedded word”. Young readers have had few occasions to develop an orthographic lexicon due to their low level of print exposure. “Embedded words” like roots are often more frequent and more familiar than derived words. Additionally, “embedded words” are easier to decode than long words, improving the opportunity to be memorized. Thus, children who have not yet developed a large orthographic lexicon benefit more from the presence of familiar “embedded words” included in long words. Thus, our study strongly indicates that morpheme units play a key role in the first stage of word identification in learning to read. This conclusion aligns with interpretations from [87,88,89,90], which suggest that “morphological representations become more independent of orthography with increasing reading ability and word exposure”.

These findings may have important implications for education. If focusing on the morphemes of a word helps children without reading disorders identify words during challenging perceptual tasks, then students who struggle with reading may also benefit from paying attention to words’ morphemic structure and to morphemic representations in written words. This strategy may provide a targeted intervention to support their reading development (e.g., [91]).

## Figures and Tables

**Table 1:** Word identifications (in percentage) as a function of stimulus type, length, fixation position, and age (Experiment 1).

	Suffixed Word					Control Word				
Stimulus	P1	P2	P3	P4	P5	P1	P2	P3	P4	P5
ADULTS										
Short word	75.6	89.6	83.6	50	24.8	78.8	92	91.6	66.8	30.4
Long word	80.8	93.6	70.8	23.2	6.8	60.8	90	72.4	30	1.6
GRADE 4										
Short word	66.7	86.2	80	64.8	35.7	56.2	73.8	77.1	67.1	44.8
Long word	45.2	67.1	52.9	31.9	15.2	28.6	46.2	45.7	26.7	13.3
GRADE 2										
Short word	77.9	91.6	87.4	68.4	42.6	68.9	88.9	88.9	77.4	48.4
Long word	63.2	87.4	76.3	44.2	14.7	43.7	66.8	73.2	50.5	18.9

**Table 2:** Word identifications (in percentage) as a function of stimulus type, fixation position, and age (Experiment 2).

	Fixation Position		
Stimulus	P1	P3	P5
ADULTS			
Suffixed words	72.73	86.67	25.76
Pseudo-suffixed words	76.58	83.03	20.00
Control words	83.03	83.94	20.61
GRADE 4			
Suffixed words	49.03	71.61	32.90
Pseudo-suffixed words	59.14	79.36	29.54
Control words	57.63	83.66	26.67
GRADE 2			
Suffixed words	29.26	54.44	21.11
Pseudo-suffixed words	52.09	72.96	24.44
Control words	45.44	71.11	20.74

## Data Availability

The data presented in this study are available on request from the corresponding author due to confidentiality and privacy disclosure considerations pertaining to the participants’ identities and authorized sharing of the data.
